# Sickle cell health awareness, perspectives and experiences (SHAPE) survey: perspectives on sickle cell disease (SCD) from healthcare providers in India, Malaysia, and Thailand

**DOI:** 10.1186/s13023-025-03765-6

**Published:** 2025-06-03

**Authors:** Roy Gomez, Annabel Su

**Affiliations:** 1 Pfizer Pte Ltd., 80 Pasir Panjang Rd, #16-81/82 Mapletree Business City, Singapore, 117372 Singapore; 2 Ipsos Healthcare, London, UK

**Keywords:** Sickle cell disease, Quality of life, Healthcare professionals, Asia

## Abstract

**Background:**

Sickle Cell Health Awareness, Perspectives and Experiences (SHAPE) Survey was previously developed for healthcare professionals, caregivers and patients from North America, Europe and Middle East. The current survey aims to broaden our understanding of the burden of Sickle Cell Disease (SCD), highlights the experiences of healthcare providers (HCPs) and identify unmet needs from Southeast Asia.

**Methods:**

The survey was a cross-sectional quantitative study conducted by Ipsos UK on behalf of Pfizer. It involved a 12-minute online survey with HCPs from India, Malaysia, and Thailand if they were managing ≥ 3 patients with SCD for > 1 year. The survey covered aspects such as the impact of SCD on quality of life, treatment, and HCP knowledge and perceptions. The responses were collected either online or through face-to-face meetings.

**Results:**

The survey link was accessed by 205 HCPs across the three countries. Of these, 105 (51.2%) HCPs were found to be eligible. A total of 85 (80.9%) HCPs completed the survey. Fatigue/tiredness and vaso-occlusive crisis (VOCs) pain were the most commonly stated symptoms by the patients. HCPs believed that SCD impacts long-term health consequences, self-esteem, and mental health. Hydroxyurea was reported to be the most commonly used treatment. The Financial impact of SCD on patients was reported by 78% HCPs. More HCPs in India consider the cost/benefit ratio to the patient and healthcare system before recommending a treatment.

**Conclusion:**

There is a need for greater recognition of the financial burden and impact on overall wellbeing of patients and their families. Surveyed HCPs are optimistic about using novel treatment options but need additional support for educating patients.

**Supplementary Information:**

The online version contains supplementary material available at 10.1186/s13023-025-03765-6.

## Introduction

Sickle cell disease (SCD) is an inherited group of blood disorders affecting the red blood cells (RBCs). The haemoglobin in the RBCs of patients with SCD is abnormal, causing the RBCs to become rigid and sticky to vascular endothelium and “sickle” shaped [1]. The root cause for the abnormal behavior of haemoglobin is due to a mis-sense mutation in the haemoglobin Subunit Beta (HBB) gene encoding the β-globin subunit of haemoglobin on the short arm of chromosome 11 [2, 3]. There are various forms of SCD including HbSS, HbSC, and HbS beta thalassemia [4]. People born with SCD exhibit symptoms from early childhood, although some children have few symptoms and lead normal lives most of the time. The common manifestations include intermittent vaso-occlusive crises, an increased risk of serious infections, hemolytic anemia and chronic pain. Acute complications associated with SCD are acute chest syndrome, acute pain episodes, respiratory failure, neurological complications and increase in volume of platelets and white blood cells. Additionally, some patients also experience other problems such as delayed growth, strokes, and lung problems [3, 5]. Sickled RBCs are highly adhesive and cause occlusion of blood vessels in nearly every organ which subsequently leads to organ damage and chronic haemolytic anemia [3].

In 2021, There were almost eight million people were living with sickle cell disease globally. Globally, the number of births of babies with sickle cell disease increased by 13.7% between 2000 and 2021. Across super-regions, sickle cell disease incidence and prevalence increased noticeably in sub-Saharan Africa; with an all-age prevalence of 5.68 million cases (4·78–6·62) in 2021, a 67.4% (62·9–71·5) increase since 2000. Over half a million babies were born with SCD in 2021 with more than 75% of them were born in sub-Saharan Africa. Furthermore, in south Asia and sub-Saharan Africa in 2021, total deaths for all ages were reported to be almost 67-times and 9-times higher, respectively, than cause specific deaths in patients with SCD [6]. There is insufficient published data from Asia in general. Although there is research published from India, the true extent of the burden of disease, its impact on the populations, and unmet needs is not well documented.

India is the second most affected nation in SCD births. In India, SCD predominantly occurs in indigenous (tribal) people, whose population is 104 million and constitutes 8.6% of India’s total population [7]. Past studies from India have reported that 20% of children with SCD die before the age of 2 [8]. A recent systematic review reported the prevalence of SCD in India to be ~ 100/100,000 preceded by Middle East and Africa. It was confirmed that sub-Saharan and North-East Africa, India, the Middle East, and the Caribbean islands are global SCD hotspots [9]. A study by Piel et al. surveyed the global distribution of neonates with sickle cell trait (SCT) (HbAS) or homozygous SCD (HbSS) revealed that high frequencies (> 10%) of the HbS allele (i.e., HbAS plus HbSS) were almost exclusively found in sub-Saharan Africa, with additional relatively high frequencies found in India [10]. There is a lack of published data from the Asia on the unmet needs of SCD from an healthcare provider (HCP) perspective.

The Sickle Cell Health Awareness, Perspectives, and Experiences (SHAPE) survey was first developed in 2021 to improve the understanding of the burden of SCD. It was developed by Global Blood Therapeutics (GBT) in partnership with SCD experts, including patient association representatives (both patients and caregivers) and HCPs. Ipsos UK, conducted the survey on behalf of GBT. This multinational survey was administered to healthcare professionals (HCPs), patients and caregivers from USA, Germany, Brazil, France, UK, Canada, Saudi Arabia, and United Arab Emirates. Additionally, patients were also surveyed from Bahrain, and Oman [11, 12].

The present study was developed to better understand and share the experiences of patients impacted by SCD in India, Malaysia, and Thailand from an HCP perspective. Furthermore, the survey also aimed to broaden the understanding of the impact of the condition and the unmet needs of those affected and to understand the inequities faced by the SCD patient community from HCPs’ perspective.

## Methods

This is a cross-sectional quantitative study, conducted by Ipsos UK on behalf of Pfizer.

The questions from the previous SHAPE survey were reviewed for suitability for India, Malaysia, and Thailand and minor revisions undertaken. The resulting survey covered the following aspects associated with SCD from the perspective of the HCPs: impact on quality of life/inequities faced by SCD patients including financial burden; treatment; and HCP knowledge & perceptions of SCD.

HCPs from India, Malaysia, and Thailand who are responsible for treating SCD were invited to take part in a 12-minute online survey (Supplement).

The survey link was shared with the HCPs who were recruited using a proprietary vendor panel and pre-qualified via a set of screening questions. Upon meeting the criteria and providing written informed consent, they were given the option to complete the survey. Participants who completed the survey were compensated for their time as per the fair market value (FMV). The inclusion criteria were: (1) practicing as hematologist, haem-oncologist, pediatrician, pediatrician hematologist, general practitioner or internist; (2) with at least 3 patients with SCD under their care; (3) practicing medicine for 3–35 years and (4) managing patients with SCD for > 1 year. Fieldwork took place from 25th July 2023 to 16th August 2023.

The survey was administered in three languages: English (India), Malay (Malaysia), and Thai (Thailand) and all the three version were equivalent in their interpretation. The survey responses were collected by interviewing HCPs either online or through face-to-face meetings with a moderator inputting response on the HCP’s behalf into the online survey. All the responses were selected from a prompted list of options shown to them. No questions asked were open ended or required unprompted answers.

An additional quality step was undertaken for including the appropriate survey responses and exclude the ones which did not pass the quality check. Only completed surveys were analysed manually and responses from those completing the questionnaire in under 5 minutes were removed if the quality of their data was poor, or if the answers did not make sense for the question. The results were analysed using quantitative analysis techniques (e.g., frequencies, mean scores) where a rating scale was used. “Top two box scores” (in which the top two responses are combined to give a single score) were analysed to summarize the data. Results were analysed at country level and in total, using Microsoft Excel. Descriptive and directional differences between countries are highlighted but are only illustrative in nature due to small sample sizes.

## Results

The survey link was accessed by 205 HCPs across the three countries. Of these, 105 (51.2%) HCPs were found to be eligible. A total of 85 (80.9%) HCPs completed the survey. All completed surveys passed the quality check. The survey was answered by 85 HCPs of which 30 HCPs were from India (35.3%), 25 HCPs were from Malaysia (29.4%), and 30 HCPs were from Thailand (35.3%).

The HCPs comprised of hematologists (28%), haem-oncologists (8%), pediatricians (22%), pediatric hematologists (18%), general practitioner/family practitioner (12%), internists (9%), and other specialties (2%). On an average, the participating HCPs had 16.8 years of experience with managing SCD patients. Further details of the participating HCPs are summarized in Table 1.

**Table 1:** Characteristics of participating HCPs

	TOTAL (*n* = 85)	India (*n* = 30)	Malaysia (*n* = 25)	Thailand (*n* = 30)
Specialty breakdown				
Haematologists	24 (28%)	5 (17%)	7 (28%)	12 (40%)
Haem-oncologists	7 (8%)	n/a	2 (8%)	5 (17%)
Paediatricians	19 (22%)	13 (43%)	4 (16%)	2 (7%)
Paediatric haematologists	15 (18%)	5 (17%)	3 (12%)	7 (23%)
General Practitioner / Family Practitioner	10 (12%)	3 (10%)	4 (16%)	3 (10%)
Internist	8 (9%)	2 (7%)	5 (20%)	1 (3%)
Other	2 (2%)	2 (7%)	n/a	n/a
**Average number of years managing patients with SCD (years)**	16.8	19.3	16.0	14.8
**Average number of patients with SCD currently under their care**	18.3	41.5	6.7	4.7
• Average number of patients with SCD aged: **11 years or younger** (mean)	5.2	12.9	1.4	1.0
• Average number of patients with SCD aged: **12–17 years old** (mean)	5.7	13.2	2.0	1.1
• Average number of patients with SCD aged: **18 years old and above** (mean)	6.4	12.9	3.3	2.5

### Impact on quality of life/inequities

As per the surveyed HCPs, for those managing patients aged 11 years or younger, fatigue/tiredness was the most common symptom reported by patients during routine visits, followed by vaso-occlusive crises (VOCs) followed by pain and yellowing of eyes/nails/skin. Among HCPs managing patients aged 12 to 17 years, VOCs pain (designated as the pain which occurs when sickled red blood cells block blood flow to the point that tissues become deprived of oxygen) was considered the most common symptom followed by fatigue/tiredness and poor appetite. For HCPs managing aged ≥ 18 years, fatigue/tiredness was reported as the most common symptom by the participating HCPs, followed by VOC pain and bone aches. Table 2 summarizes the responses from HCPs based on the list of 19 symptoms presented to them to select from.

**Table 2:** Top three most common stated symptoms by patient age groups

Age Group	Total	India	Malaysia	Thailand
Patients 11 years of age or under	Fatigue / tiredness (84%)	VOCs Pain (89%)	Fatigue / tiredness (100%)	Fatigue / tiredness (100%)
	VOCs Pain (80%)	Fatigue / tiredness (70%)	Poor appetite (82%)	Headache (100%)
	Yellow eyes / nails / skin (68%)	Bone aches (67%)	Generalised pain (82%)	Generalised pain (92%)
				Yellow eyes / nails / skin (92%)
Patients aged 12–17	VOCs Pain (92%)	VOCs Pain (96%)	Fatigue / tiredness (95%)	Fatigue / tiredness (95%)
	Fatigue / tiredness (89%)	Fatigue / tiredness (81%)	VOCs Pain (89%)	Generalised pain (95%)
	Poor appetite (77%)	Yellow eyes / nails / skin (73%)	Poor appetite (89%)	VOCs Pain (90%)
		Bone aches (73%)	Generalised pain (89%)	Poor appetite (90%)
				Yellow eyes / nails / skin (90%)
Patients aged 18 years or above	Fatigue / tiredness (93%)	Fatigue / tiredness (84%)	Fatigue / tiredness (100%)	VOCs Pain (100%)
	VOCs Pain (93%)	VOCs Pain (84%)	Headache (100%)	Fatigue / tiredness (95%)
	Bone aches (90%)	Bone aches (84%)	VOCs Pain (94%)	Generalised pain (95%)
		Headache (68%)	Bone aches (94%)	Poor appetite (95%)
		Low mood / feeling down / depressed (58%)	Yellow eyes / nails / skin (94%)	Bone aches (90%)
		Yellow eyes / nails / skin (58%)	Generalised pain (94%)	Low mood / feeling down / depressed (90%)
		Signs of organ damage (58%)	Nausea (94%)	Vision difficulties / retinopathy (90%)
		Leg ulcers (58%)		Poor sleep (Insomnia) (90%)

Base All respondents: Patients 11 years of age or under: Total *n* = 50, India *n* = 27*, Malaysia *n* = 11*, Thailand *n* = 12*

Patients aged 12–17: Total *n* = 65, India *n* = 26*, Malaysia *n* = 19*, Thailand *n* = 20*

Patients aged 18 years or above: Total *n* = 58, India *n* = 19*, Malaysia *n* = 18*, Thailand *n* = 21*

*Small base size

While VOC pain, bone aches and fatigue were the most common SCD symptoms reported across age groups; adult patients were more likely to report a range of symptoms including mental health (low mood/feeling down/depressed was reported by 79% of patients aged ≥ 18 years vs. 20% and 57% of patients aged < 11 years and 12–17 years, respectively).

The survey assessed impact of SCD on various aspects of life in patients below 18 years of age. The surveyed HCPs managing these patients believed that long-term health prospects (58%), self-esteem (45%) and mental health (45%) were the most impacted parameters.

**Fig. 1:**
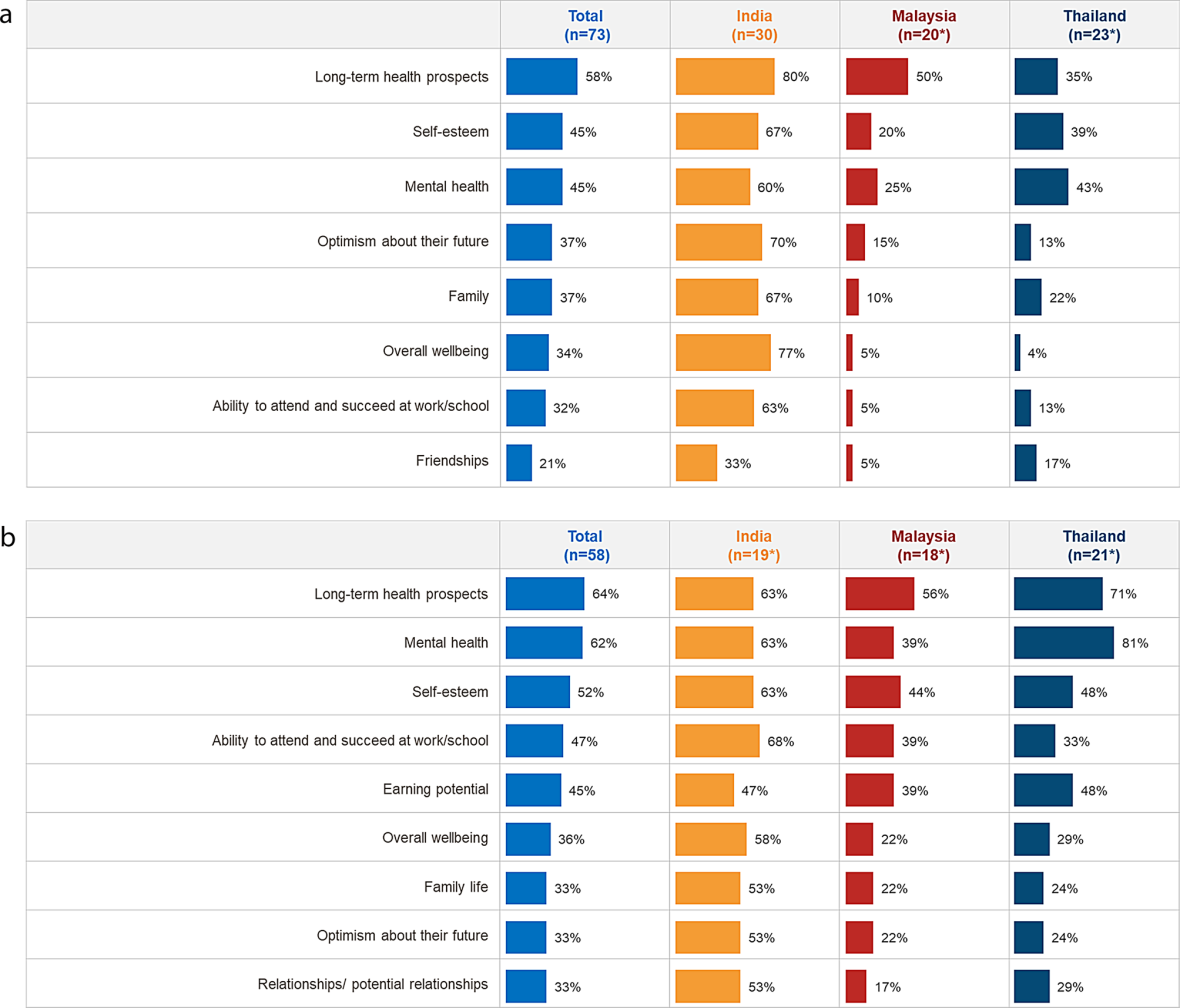
**a** Impact of SCD on younger patients’ (aged under 18) lives. **b** Impact of SCD on adult patients’ (aged above 18) lives. Impact of SCD on patients, by age. Figures show % of HCPs selecting ‘to some extent’ or ‘to a great extent’. *Small base size

Among the HCPs managing patients with SCD aged under 18 years, Indian HCPs indicated long-term health prospects, overall wellbeing, and optimism about their future as the most impacted, while HCPs from Malaysia and Thailand considered long-term health prospects and mental health to be the most impacted aspects in the lives of patients with SCD (aged < 18) (Fig. 1a). It was observed that the same areas were also impacted in adult patients with SCD across three countries with the additional effect on patient ability to work and earning potential. The parameters impacted for adult patients were similar across the three surveyed countries (Fig. 1b).

**Fig. 2:**
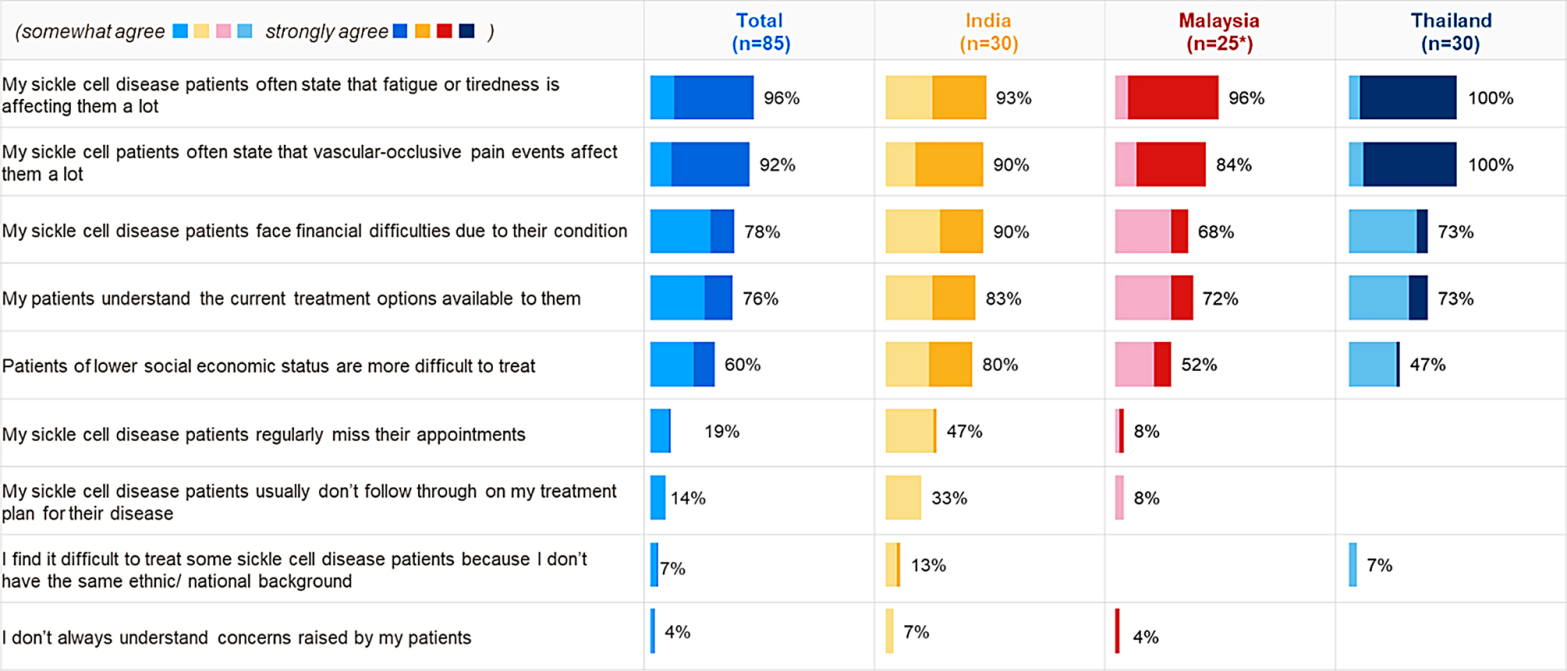
HCPs’ attitudes to SCD patient management. Figures show % of HCPs selecting ‘somewhat agree’ or ‘strongly agree

In addition to the symptoms of SCD, financial difficulties associated with SCD was evident in all three countries (Fig. 2), affecting both patients and HCPs. For Indian HCPs, patient compliance with both appointments (47%) and treatments (33%) were of higher concern. Financial impact of SCD is evident across three countries, with 78% of surveyed HCPs agreeing that their patients with SCD face financial difficulties.

### Available treatment options and challenges

**Fig. 3:**
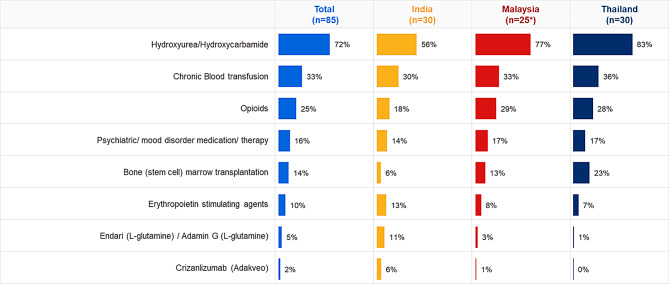
Proportion of patients with SCD on treatments (mean %)

Hydroxyurea/hydroxycarbamide was the main treatment for patients (72% overall; 56% India, 77% Malaysia, and 83% Thailand). Around one-third HCPs (33%) indicated use of chronic blood transfusions in all three countries (30% India, 33% Malaysia, 36% Thailand). Opioids were indicated to be a part of the management plan by 25% HCPs (18% India, 29% Malaysia, 28% Thailand). Other management options (Fig. 3) are not as widely used.

Overall, all HCPs surveyed agreed that early treatment of sickle cell disease can help with managing/preventing severe symptoms. Further, 86% HCPs (77% from India, 88% from Malaysia, and 93% from Thailand) believed that treating haemolytic anaemia can improve most aspects of SCD.

**Fig. 4:**
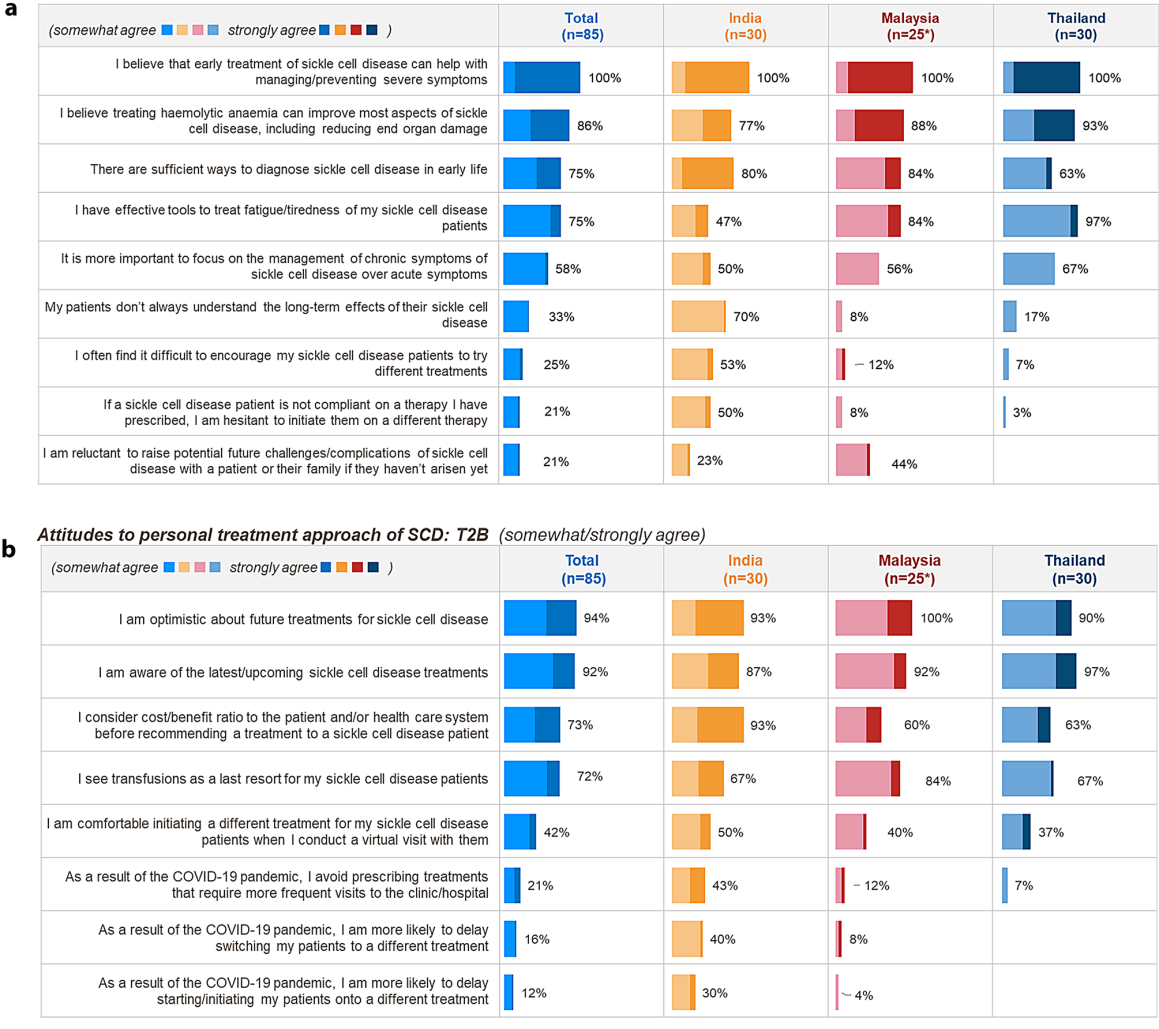
**a** Attitude towards overall treatment. **b** Attitudes to personal treatment approach of SCD. HCP attitudes to treatment approach. Figures show % of HCPs selecting ‘somewhat agree’ or ‘strongly agree’

HCPs in India reported that they faced difficulties in treatment of SCD, in terms of patients not always understanding the long-term effects of their sickle cell disease (70%) and difficulty in encouraging patients to try new treatments (53%). In contrast, these concerns were not reported strongly by Malay and Thai HCPs. Figure 4a summarizes the attitude of surveyed HCPs regarding SCD management.

Our results also demonstrated that 94% of the HCPs felt optimistic about the future of SCD management. It is important to note that 92% of HCPs from the participating countries claimed to be up to date in the latest SCD treatments.

Cost of treatment was a strong influential factor for treatment decisions in India, with more HCPs in India (93%) considering the cost/benefit ratio to the patient and/or healthcare system before recommending a treatment vs. Malaysia (60%) and Thailand (63%). (Refer Fig. 4b)

Surveyed HCPs from all three countries ranked previous experience of side effects and inconvenient dosing regimen to be the most common reasons for refusing treatment in general by the patients.

**Fig. 5:**
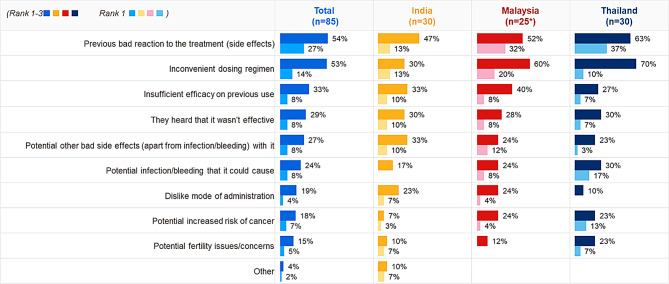
Main reasons patients with SCD refuse treatment. *Figures show % of HCPs ranking statements 1–3 and Rank 1*

Potential risk of infection or bleeding was an additional concern reported by Thai HCPs. (Fig. 5).

### HCP knowledge and perceptions of SCD

**Fig. 6:**
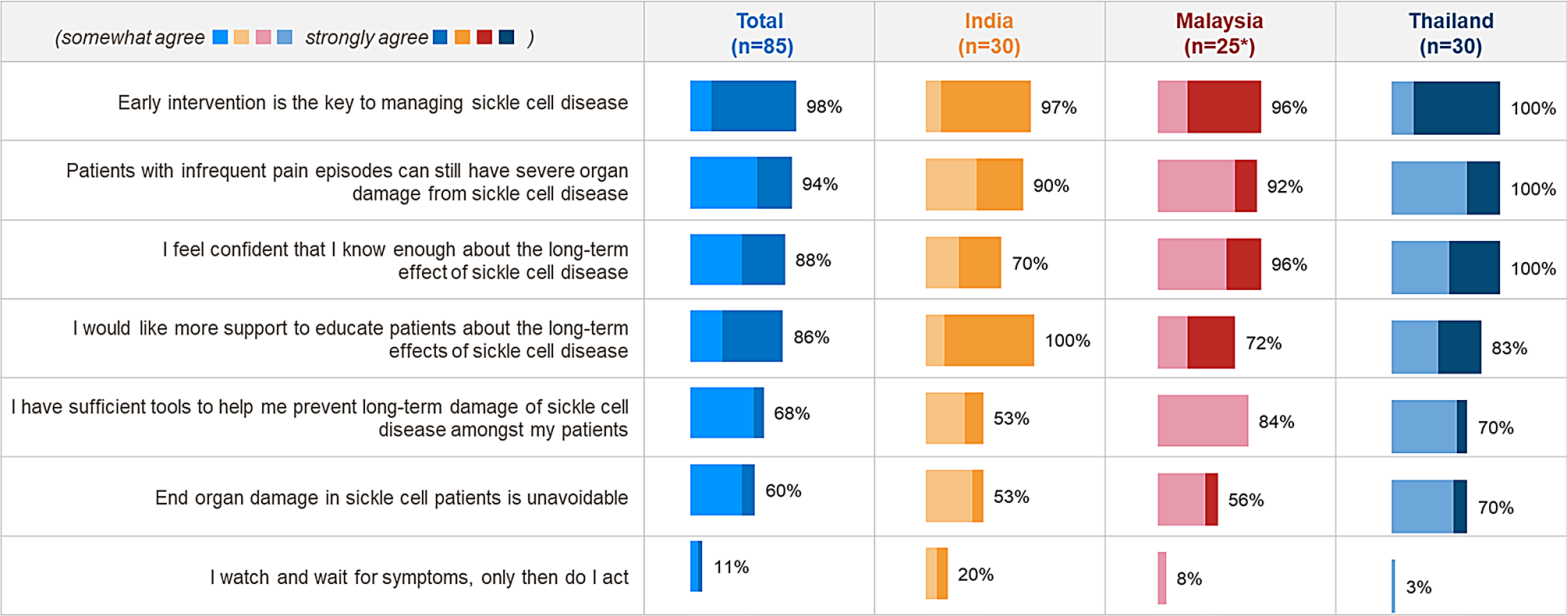
HCP Knowledge and perceptions of SCD management. Figures show % of HCPs selecting ‘somewhat agree’ or ‘strongly agree’

Majority (98%) of the surveyed HCPs agreed that early intervention is key to effective SCD management across all three countries. Around 94% HCPs agreed that patients with infrequent pain episodes can still have severe organ damage from sickle cell disease (Fig. 6).

**Fig. 7:**
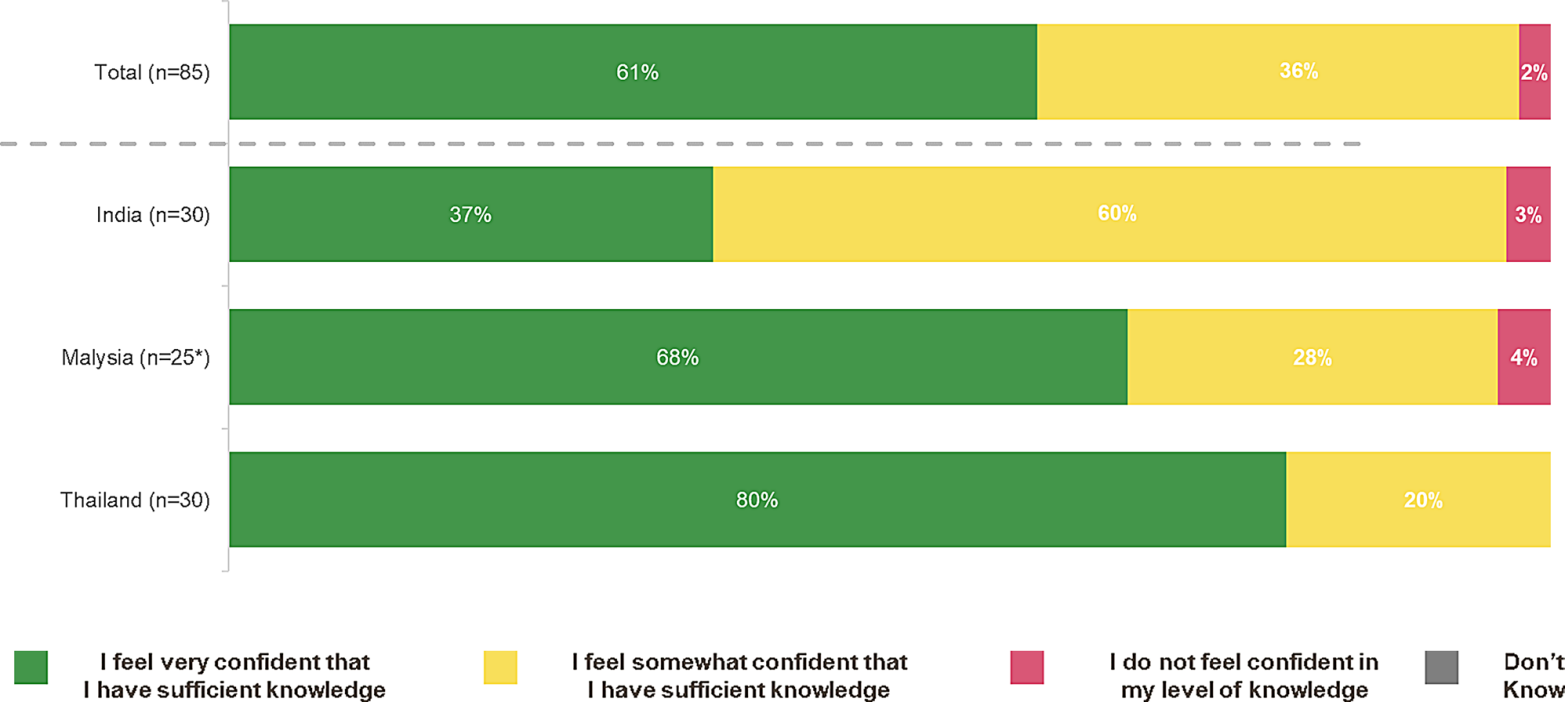
HCP Knowledge of the consequences of haemolytic anaemia and haemolysis (%)

Overall, 61% HCPs felt confident about their knowledge on haemolytic anaemia and haemolysis. It was noted that 80% HCPs from Thailand, 68% HCPs from Malaysia, and 37% HCPs from India felt confident in their knowledge on haemolytic anaemia and haemolysis (Please see Fig. 7).

**Fig. 8:**
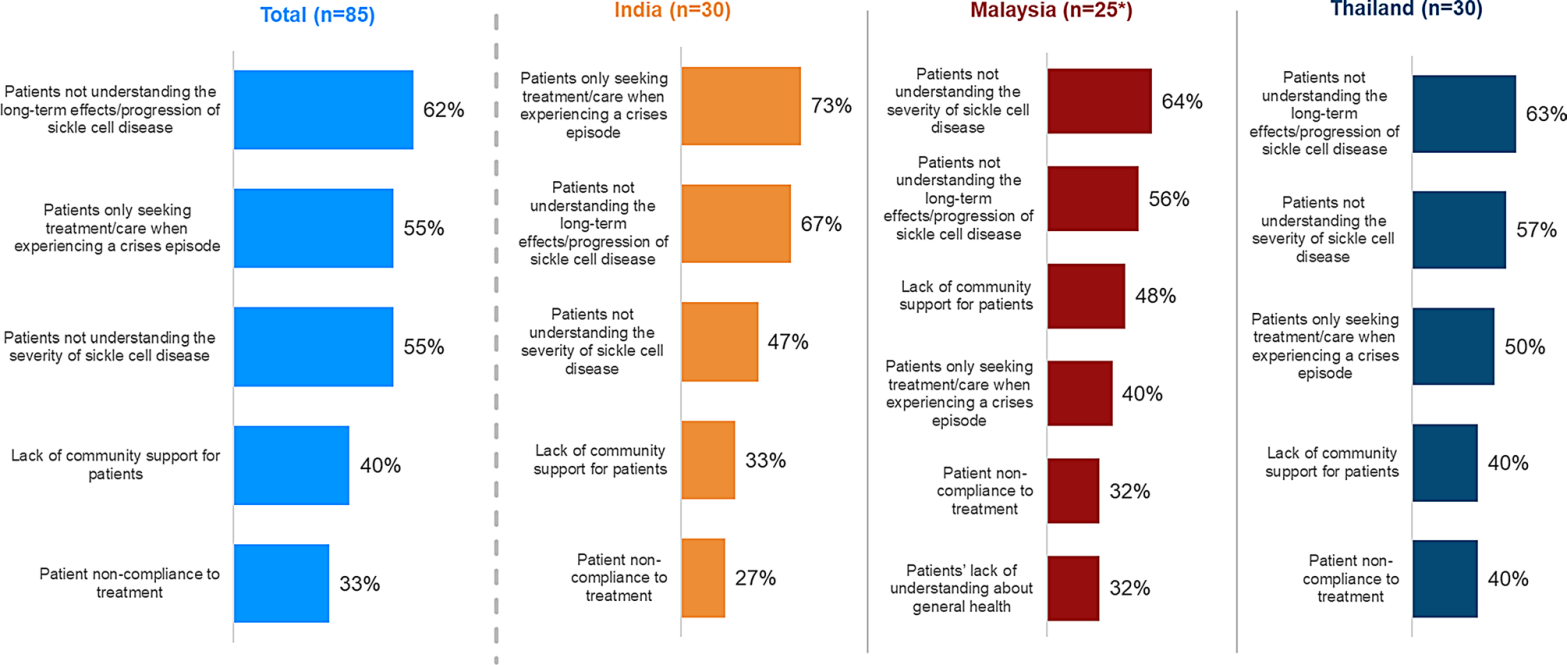
HCPs’ top five concerns for patients with SCD (%)

Patients not understanding the long-term effects or the progressive nature of SCD was reported as the topmost concern for patients as per 62% of the participating HCPs. Lack of patient understanding of severity (55%) and patients seeking medical treatment/care when experiencing a crises episode (55%) were also noted as concerns for patients with SCD across three countries. Other concerns include lack of community support (40%) and patient non-compliance to treatment (33%) (Please see Fig. 8 for country-wise breakup).

The surveyed HCPs were asked to indicate the main source of their information on SCD. Overall, 82% HCPs have received information from conferences followed by national or regional guidelines (69%). Other sources of information included colleagues (59%), pharmaceutical website (52%), and recommendations from international medical societies (48%). Table 3 summarizes the country-wise top sources of information utilized by HCPs.

**Table 3:** Top five most common sources of information about SCD (%)

Source	Total (*n* = 85)	India (*n* = 30)	Malaysia (*n* = 25)	Thailand (*n* = 30)
Conferences	82%	80%	88%	80%
National or regional guidelines	69%	90%	52%	63%
Colleagues	59%	37%	72%	70%
Pharmaceutical websites	52%	7%	76%	77%
Recommendations from international medical societies	48%	73%	36%	33%

## Discussion

In this survey, HCPs from Malaysia, Thailand, and India indicated that pain and fatigue are the most significant symptoms for SCD patients. Younger patients in Malaysia and Thailand often reported poor appetite and generalized pain, while adults commonly experienced bone aches. Adults faced more extensive physical and mental health impacts, whereas younger patients were affected by self-esteem issues and long-term mental health.

Indian HCPs highlighted the substantial impact of SCD on long-term health prospects and overall well-being, especially among younger patients. Financial challenges also affect patient care across all three countries. HCPs agreed that early treatment can manage severe symptoms and improve various aspects of SCD.

Patient non-compliance due to difficulty staying in treatment was a key issue, particularly in India where there is a lack of awareness and resources for effective management. HCPs in Malaysia and Thailand reported better awareness and treatment resources but still see room for improvement with new treatments. Conferences and national guidelines are key information sources for Indian HCPs, while Malay and Thai HCPs rely on conferences and pharmaceutical websites.

In South Asia, the highest prevalence of the SCD is observed in India. The prevalence of the disease in India is estimated to be second to that of Africa with the highest frequency of βs allele [13]. Despite the high burden of SCD in India, there are no state-led public health programmes, and limited interventions for treatment and management of SCD are available [14]. There are efficacious interventions available to manage SCD which are available at government run healthcare centers. But the treatments do not always reach patients with SCD. There are no structured referral system and standard treatment guidelines for managing SCD. The Government of India has formulated guidelines for the prevention and control of haemoglobinopathies in India [15].

SCD has been predominantly identified in tribal/indigenous communities in India. These regions lack the required resources and facilities to diagnose and manage patients with SCD [7, 16]. Although, research has been conducted on SCD in India, there are limited number of studies which are mostly microstudies and screened people for genetic and anthropological research. Access to clinical and patient management data is much needed and will help inform and improve the management of SCD in India [7]. There are a few population based Indian studies conducted with a geographically limited cohorts [16–20]. A scoping review of published SCD studies from India revealed that there are significant gaps in comprehensiveness of research particularly concerning design, geographical scope, implementation, or systematic appraisal of quality of care for patients with SCD. There are no long-term studies conducted which critically limits the availability of evidence of healthcare outcomes in SCD. Most research highlights health services or innovations provided in NGO settings with few studies analyzing government health services’ response to SCD at regional/national level [21]. Furthermore, it has been speculated that the prevalence previously reported could be under-reported and the burden could be more than anticipated [22].

India has made efforts towards SCD screening programs, delivery of hydroxyurea therapy and bone marrow transplantation because of the increased investments over the past several years [22]. The proposed Indian SCD registry (ISCDR), is part of an ongoing intervention meant to develop a comprehensive model of screening and management of SCD by the primary health centers (PHCs) or community health centers (CHCs) in six tribal areas: Chhoteudepur (Gujarat), Mysuru (Karnataka), Anuppur (Madhya Pradesh), Kandhamal (Odisha), Visakhapatnam (Andhra Pradesh) and Udalguri (Assam). If implemented, this registry will facilitate systematic longitudinal collection of demographics, epidemiological, health services and treatment-related data, required for planning research and health policies to manage SCD effectively. The services can be scaled up to all SCD-endemic districts throughout the country [7].

A survey by Das et al., 2023 assessed knowledge and perception related to SCD among tribal community from Odisha, India. They found that many of the participants were aware about SCD, its clinical signs/symptoms, and about the modern medication for SCD treatment and their benefits. But very few participants were aware about the cause of SCD, the importance of treatment adherence, and uncommon symptoms of SCD. However, it was also found that since the medications and treatment for SCD are not available in the nearby PHCs, patients have to travel long distances and incur out of pocket expenditure in order to avail the basic treatment which has led to failure from treatment adherence in many cases. Hence the patients prefer to go for treatment only during crisis [23]. Another survey reported the extent of SCD-related knowledge and management practices of peripheral health workers located in tribal areas of India. It was found that they have inadequate knowledge about SCD- and management practices [16]. This further highlights the need for generating awareness not only among at-risk populations but also HCPs and allied health workers to improve SCD management in the country.

There is lack of sufficient literature from Thailand and Malaysia on SCD burden and management. A case series by Xu et al., 2019 identified for the first time the origin of a β^S^ allele in Thailand. They attributed population migration to be cause for expansion of the β^S^ mutation into previously nonendemic regions and to the establishment of the mutation in new populations through genetic admixture. The study also highlighted that there are existing thalassemia prevention and control programme but there is a need to develop a regional or national newborn screening programme for haemoglobinopathies including SCD [24]. This implies that there is low burden of identified SCD in Thailand but due to migration and intermarriage, the burden of SCD is expected to increase.

Previous SHAPE surveys from USA, European countries and Middle Eastern countries have been presented as abstracts at international congresses. These surveys have reported challenges pertaining to understanding patient needs, and thus the need for additional support to communicate with patients and educate them. HCPs surveyed also noted an unmet need for treatments that fully address end-organ damage in SCD. Furthermore, the survey revealed that HCPs face a complex environment when treating patients with SCD due to differences in socioeconomic status and ethnic background [12]. The survey on patients and caregivers concluded that most patients seek to reduce risk of long-term complications and haemolytic anaemia. The symptoms experienced by patients with SCD impact the patient as well as the caregivers who face challenges like fatigue and loss of earnings. The authors highlighted need to increase support to improve the situation for patients as well as caregivers [11]. The Gulf subset additionally revealed the extent of physical and emotional burden of SCD on patients and caregivers. Caregivers in Gulf countries expressed concerns about early loss of life and worsening SCD symptoms. These findings stressed on the need for effective treatments that alleviate symptoms and manage disease sequalae, and for additional resources to improve the quality of life of patients and caregivers [25]. The recently published SHAPE survey article by *de Montalembert et al.*., shared the findings of a similar survey questionnaire taken by patients, caregivers and HCPs from the USA, Brazil, the UK, France, Saudi Arabia, the UAE, Canada, Bahrain, Germany, and Oman. The HCP survey results highlighted fatigue/tiredness, VOC pain, and bone aches as most common symptoms reported by their patients. The majority of the HCPs agreed that SCD has a significant impact on patients’ overall well-being, long-term health outcomes, and future outlook. Most HCPs agreed that early treatment of SCD can help prevent or manage serious symptoms and that treating haemolytic anaemia can improve many aspects of SCD, including reducing organ damage. However, only 53% of HCPs felt they have effective tools to treat this common symptom, and 53% had difficulty persuading their patients to try different treatments. The most common treatments for SCD reported by HCPs were hydroxyurea, opioids, and regular blood transfusion. Although, majority of the HCPs were confident about their knowledge of the long-term effects of SCD, they wanted more support to educate their patients about these long-term effects [26]. The findings related to most common symptoms, impact of patients’ lives, attitude towards overall treatment, and perception of SCD management were similar to the observations of the current survey. Although hydroxyurea was also the most frequently used treatment in all three countries in the current survey, only 43% HCPs in the survey published by *de Montalembert et al.* [26] reported use of hydroxyurea. There was a disparity among the three countries in terms of HCP knowledge. The overall proportion of HCPs positively reporting their confidence about their knowledge, it was less as compared to HCPs surveyed by *de Montalembert et al.* [26].

The study was limited by the small sample size, which may have limited the generalizability of the findings. The surveyed population may not fully represent the diverse demographics and varying healthcare settings within the three countries. However, there was a diverse representation from various specialties within the country.

## Conclusions

There is need for generating local and culturally specific solutions to address the increasing concern of SCD in the region. Some of the ways to address the unmet needs could be: implementation of national level societies and registries; promote skill building for healthcare workers, training HCPs and healthcare professionals; initiatives from private and public sectors to improve access to hydroxyurea, bone marrow transplant, and other treatment options; family counselling, promoting advocacy and awareness, nutrition and psychosocial support, support for transitioning from pediatric to adult care, integrative/complementary strategies will further improve patient outcomes.

The challenges for SCD management are diverse within the three surveyed countries in Asia. There is a need for greater recognition of the financial burden and impact on overall wellbeing of patients and their families as well as working towards better delivery of clinical care. Surveyed HCPs are optimistic about using novel treatment options but need additional support for educating patients.

## Electronic supplementary material

Below is the link to the electronic supplementary material.

Supplementary Material 1

## Data Availability

The authors confirm that the data supporting the findings of this study are available within the article. Additional data recording individual responses to the survey are available on request from the corresponding author, Roy Gomez. The data are not publicly available due to as it contains personal identifiers which could compromise the privacy of survey participants.
