# Supplementary material for: Sickle cell health awareness, perspectives and experiences (SHAPE) survey: perspectives on sickle cell disease (SCD) from healthcare providers in India, Malaysia, and Thailand
Source: Orphanet J Rare Dis. 2025 Jun 3;20:267. doi: 10.1186/s13023-025-03765-6 (PMC12131614; doi:10.1186/s13023-025-03765-6)
Supplement: Supplementary file 1 — Supplementary Material 1 [file 13023_2025_3765_MOESM1_ESM.docx]

**Supplement**

**Sickle Cell Disease Survey Questions**

| **Sr. No.** | **Question** | **Answer** |
| --- | --- | --- |
|  | Do you have a dedicated ‘sickle cell disease (SCD)’ clinic? | Yes  No |
|  | What all facilities are provided at your clinic? | Newborn/prenatal screening facilities  Diagnostic facilities  Genetic counselling  Family counselling  Crisis management  Blood/Bone marrow transplantation  Psychological counselling  Vaccination to prevent vaccine preventable diseases (VPDs) |
|  | How many SCD patients are you currently treating? |  |
|  | How many patients are diagnosed with SCD per year? |  |
|  | How are the patients identified? | Patients consult on their own/Patients directly approach the specialist  Referred by primary care physicians  Referred by nurses  Referred by friends/family  Others, please specify: ___________ |
|  | What is the catchment area for majority of the patients? | Tier I cities  Tier II cities  Tier III cities  Rural areas with substantial tribal population  Rural areas with primary care center  Rural areas with insufficient healthcare facilities |
|  | Which treatment options are available at your center? | Hydroxyurea  L-glutamine  Crizanlizumab  Voxelotor  Analgesics  Iron and folic acid supplements  Others, please specify: ___________ |
|  | How do you treat the patients? | Hydroxyurea  L-glutamine  Crizanlizumab  Voxelotor  Analgesics  Iron and folic acid supplements  Others, please specify: ___________ |
|  | Are the patients additionally using alternative medicine/home remedies? | Yes  If yes, please specify: ­­­­­­­­­____________  No  Unsure |
|  | How often are the patients followed-up? |  |
|  | Is the treatment compliance checked for patients being treated? | Yes  If yes, please specify how compliance is checked: ­­­­­­­­­____________  No |
|  | How many bed stays/hospitalizations are observed per year? |  |
|  | What is the average length of hospital stay among SCD patients? |  |
|  | Are the current treatment options satisfactory to address the needs of SCD patients? | Yes  If yes, please specify if any additional support can be provided: ­­­­­­­­­____________  No  If no, please state the reason: _________ |
